# Efficient interlayer charge release for high-performance layered thermoelectrics

**DOI:** 10.1093/nsr/nwaa085

**Published:** 2020-04-28

**Authors:** Hao Zhu, Zhou Li, Chenxi Zhao, Xingxing Li, Jinlong Yang, Chong Xiao, Yi Xie

**Affiliations:** Hefei National Laboratory for Physical Sciences at the Microscale, University of Science and Technology of China, Hefei 230026, China; Hefei National Laboratory for Physical Sciences at the Microscale, University of Science and Technology of China, Hefei 230026, China; Hefei National Laboratory for Physical Sciences at the Microscale, University of Science and Technology of China, Hefei 230026, China; Department of Chemical Physics, Hefei National Laboratory for Physical Sciences at the Microscale and Synergetic Innovation Center of Quantum Information & Quantum Physics, University of Science and Technology of China, Hefei 230026, China; Department of Chemical Physics, Hefei National Laboratory for Physical Sciences at the Microscale and Synergetic Innovation Center of Quantum Information & Quantum Physics, University of Science and Technology of China, Hefei 230026, China; Hefei National Laboratory for Physical Sciences at the Microscale, University of Science and Technology of China, Hefei 230026, China; Institute of Energy, Hefei Comprehensive National Science Center, Hefei 230031, China; Hefei National Laboratory for Physical Sciences at the Microscale, University of Science and Technology of China, Hefei 230026, China; Institute of Energy, Hefei Comprehensive National Science Center, Hefei 230031, China

**Keywords:** layered superlattice material, interlayer charge release, carrier concentration, thermoelectric performance

## Abstract

Many layered superlattice materials intrinsically possess large Seebeck coefficient and low lattice thermal conductivity, but poor electrical conductivity because of the interlayer transport barrier for charges, which has become a stumbling block for achieving high thermoelectric performance. Herein, taking BiCuSeO superlattice as an example, it is demonstrated that efficient interlayer charge release can increase carrier concentration, thereby activating multiple Fermi pockets through Bi/Cu dual vacancies and Pb codoping. Experimental results reveal that the extrinsic charges, which are introduced by Pb and initially trapped in the charge-reservoir [Bi_2_O_2_]^2+^ sublayers, are effectively released into [Cu_2_Se_2_]^2−^ sublayers via the channels bridged by Bi/Cu dual vacancies. This efficient interlayer charge release endows dual-vacancy- and Pb-codoped BiCuSeO with increased carrier concentration and electrical conductivity. Moreover, with increasing carrier concentration, the Fermi level is pushed down, activating multiple converged valence bands, which helps to maintain a relatively high Seebeck coefficient and yield an enhanced power factor. As a result, a high *ZT* value of ∼1.4 is achieved at 823 K in codoped Bi_0.90_Pb_0.06_Cu_0.96_SeO, which is superior to that of pristine BiCuSeO and solely doped samples. The present findings provide prospective insights into the exploration of high-performance thermoelectric materials and the underlying transport physics.

## Teaser

Taking BiCuSeO as an example, we propose an efficient interlayer charge release strategy to optimize electrical transport properties in layered thermoelectrics by simultaneously providing charges and interlayer charge-transport channels.

## INTRODUCTION

As the core component of thermoelectric generators and solid-state Peltier coolers [1–5], thermoelectric materials enable direct and reversible conversion between heat and electricity [6–9]. The conversion efficiency of thermoelectric material is quantified by the dimensionless figure of merit, *ZT* = *S*^2^*σT*/(*κ*_lat_ + *κ*_ele_), where *S*, *σ*, *T*, *κ*_lat_ and *κ*_ele_ are the Seebeck coefficient, electrical conductivity, absolute temperature, lattice and electronic components of the total thermal conductivity (*κ*_tot_ = *κ*_lat_ + *κ*_ele_), respectively [10–12]. Hence, high-performance thermoelectric materials need to meet the following criteria: (i) high electrical conductivity; (ii) large Seebeck coefficient; and (iii) low total thermal conductivity. However, for well-known reasons, improvement in *ZT* is greatly constrained by the inter-coupled electrical and thermal transport parameters [5,11,13–15]. Therefore, exploring effective strategies for decoupling these interrelated parameters is of great importance for breakthroughs in thermoelectric research [10–13].

Thermoelectric performance can be significantly improved in layered superlattice materials [16], arising mainly from an increase in Seebeck coefficient as a result of the peculiar electronic structure. Meanwhile, the comparatively weak bonding between the sublayers endows semiconductor superlattices with intrinsic low lattice thermal conductivity [17–22]. Unfortunately, the superlattice structure does not favor fine electrical conductivity in the bulk state, which has proved a stumbling block to achieve high thermoelectric figure of merit in two-dimensional superlattices [22–26]. Specifically, for a multi-layered superlattice with alternating stacked insulating sublayers and conductive sublayers, charges are mostly trapped in the insulating sublayers. The concentration of charges stored in the insulating charge-reservoir sublayers is extremely low. Furthermore, release of the dominant trapped charges into the conductive sublayers to become conduction carriers is difficult because they must surmount the interlayer energy barrier. As a result, intrinsically low carrier concentration is found in thermoelectric superlattices, accounting for the poor electrical conductivity. These issues urge us to find a novel strategy to tailor the trapping and conduction characteristics of charges in a superlattice system.

Generally, element or vacancy doping is the primary choice for regulating the carrier concentration toward an optimal range of 10^19^–10^21^ cm^−3^ [3–5]. However, as discussed above, superlattice compounds suffer from both intrinsic low charge concentration and absence of charge-transport channels, implying that the single doping is overstretched. It is therefore urgently necessary to develop multiple doping for carrier concentration optimization in thermoelectric superlattices. On the other hand, it is well known that band convergence has been demonstrated to be a robust strategy for yielding high power factor (*S*^2^*σ*) in thermoelectrics [3–5,11]. For compounds exhibiting multiple extrema with energy difference of no more than a few *k*_B_*T* in the energy bands, it is essential to move the Fermi level significantly in energy so that more Fermi pockets can be populated. However, in most cases, the intrinsic low carrier concentration is not sufficient to activate the multiple converged bands.

**Scheme 1.**
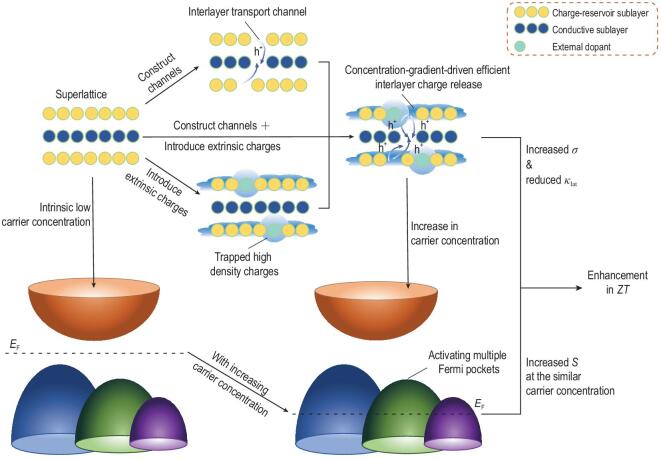
Efficient interlayer charge release (taking hole carriers as an example) activating multiple Fermi pockets in layered superlattice.

In view of the above situation, one may expect that if additional charges and interlayer charge-transport channels are provided simultaneously in thermoelectric superlattice, the carrier concentration would be significantly increased. Furthermore, the multiple converged bands could be expected to be activated as the increase in carrier concentration could regulate the position of Fermi level. We put forward the idea of efficient interlayer charge release via multiple-defect codoping, in which some defects construct channels for interlayer charge-transport process, while others provide plentiful extrinsic charges to diffuse along these channels (as shown in Scheme 1). It is expected that efficient interlayer charge release in superlattice will ensure high carrier concentration and activate multiple converged bands if there are multiple extrema in the energy band.

BiCuSeO provides an ideal platform for the above strategy. As a typical superlattice material, BiCuSeO consists of [Bi_2_O_2_]^2+^ and [Cu_2_Se_2_]^2−^ sublayers [27–31] stacking alternately along the *c* axis of the tetragonal cell. In BiCuSeO, the insulating [Bi_2_O_2_]^2+^ sublayers act as a charge reservoir, while conduction takes place in the conductive [Cu_2_Se_2_]^2−^ sublayers [27,28]. Intrinsically low electrical conductivity is found in pristine BiCuSeO [32–38], arising from the extremely low carrier concentration. BiCuSeO has a complex electronic structure with multiple extrema in the valence bands [27], but the intrinsically low carrier concentration is insufficient to allow holes to populate those multiple converged valence bands. In the present study, for the purpose of achieving efficient interlayer charge release and thereby activating multiple Fermi pockets in BiCuSeO, we demonstrate an integrated strategy through Bi/Cu dual vacancies and Pb codoping. Specifically, Bi/Cu dual vacancies construct channels for interlayer charge-transport process, and Pb-doping introduces plentiful extrinsic charges, which are initially trapped in [Bi_2_O_2_]^2+^ sublayers. As expected, our studies show that charge concentration gradient drives release of these confined charges, enabling the charges to almost completely diffuse into [Cu_2_Se_2_]^2−^ sublayers along the interlayer transport channels and thus become conduction carriers. As a result, the concentration of conduction holes is remarkably increased in vacancies/Pb codoped BiCuSeO, reaching the theoretical limiting value. This efficient interlayer charge release in Bi_1-x-y_Pb_y_Cu_1-x_SeO results in significant enhancement in carrier concentration and thus electrical conductivity. Meanwhile, the substantial increase in carrier concentration pushes the Fermi level into the valence band, activating multiple converged valence bands, which enables a relatively high Seebeck coefficient and yields an increased power factor for Bi_1-x-y_Pb_y_Cu_1-x_SeO. As a consequence, a maximum *ZT* value of ∼1.4 for Bi_0.90_Pb_0.06_Cu_0.96_SeO is derived at 823 K, which is superior to that of (i) the pristine BiCuSeO, (ii) BiCuSeO solely doped with Bi/Cu dual vacancies, and (iii) BiCuSeO solely doped with Pb. The present results open up a promising avenue for regulating transport properties in thermoelectrics.

## RESULTS AND DISCUSSION

The powder X-ray diffraction (PXRD) patterns of Bi_1-x-y_Pb_y_Cu_1-x_SeO are shown in Supplementary Fig. S1a. All samples in the present study are single phase and every reflection in the PXRD pattern can be indexed to the tetragonal *P*4/*mmm* space group of the parent BiCuSeO oxyselenides. Supplementary Fig. S1b shows a HAADF STEM image of Bi_0.90_Pb_0.06_Cu_0.96_SeO taken along the [100] zone axis, confirming the typical layered feature of the BiCuSeO compound. The EDS mappings of Bi_0.90_Pb_0.06_Cu_0.96_SeO (shown in Supplementary Fig. S1c) demonstrate the homogeneous single phase in BiCuSeO systems.

**Figure 1.**
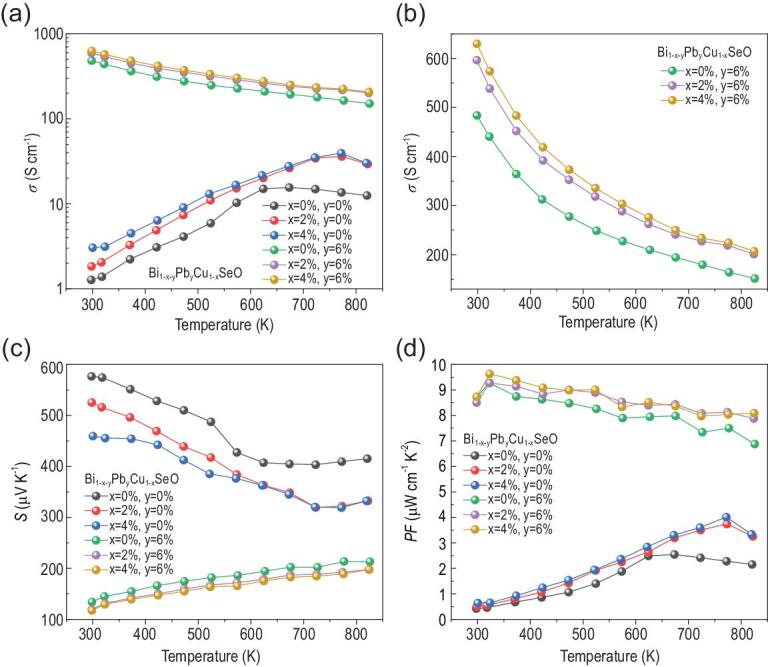
Electrical transport properties as a function of temperature for Bi_1-x-y_Pb_y_Cu_1-x_SeO: (a) electrical conductivity; (b) enlarged electrical conductivity ranging from 130 to 650 S cm^−1^; (c) Seebeck coefficient; (d) power factor.

Figure 1 plots the temperature-dependent electrical transport properties of Bi_1-x-y_Pb_y_Cu_1-x_SeO samples. With increasing doping fraction of Bi/Cu dual vacancies, the electrical conductivity increases from ∼1.3 S cm^−1^ for pristine BiCuSeO to ∼1.8 S cm^−1^ for Bi_0.98_Cu_0.98_SeO, and then to ∼3.1 S cm^−1^ for Bi_0.96_Cu_0.96_SeO at room temperature (Fig. 1a). An analogous relationship between the electrical conductivity and concentration of dual vacancies is also observed in dual-vacancy- and Pb- codoped samples (Fig. 1b). Meanwhile, for Pb-doped BiCuSeO, the electrical conductivity gradually decreases with rising temperature, exhibiting characteristics of metallic conduction and a heavily doped state. In addition, the electrical conductivity of Pb-doped samples is significantly increased compared to that of the Bi_1-x_Cu_1-x_SeO without Pb doping over the entire test temperature range. Consequently, the dual-vacancy- and Pb- codoped sample (that is, Bi_0.90_Pb_0.06_Cu_0.96_SeO) features a maximum electrical conductivity of ∼629.9 S cm^−1^ at room temperature, which is higher than that of the solely Pb-doped samples (∼483.7 S cm^−1^ for Bi_0.94_Pb_0.06_CuSeO at room temperature) and far higher than that of the solely dual-vacancy-doped samples (∼3.1 S cm^−1^ for Bi_0.96_Cu_0.96_SeO at room temperature). The transport properties for the Bi_1-x-y_Pb_y_Cu_1-x_SeO samples are listed in Supplementary Table S1, and it can be concluded that the enhancement in electrical conductivity of dual-vacancy- and Pb- codoped samples is mainly a result of the significantly increased carrier concentration.

Figure 1c depicts the Seebeck coefficients as a function of temperature for Bi_1-x-y_Pb_y_Cu_1-x_SeO samples. The positive Seebeck coefficient for all samples in the entire temperature range reflects p-type conduction, which is consistent with the conclusion given by the Hall measurement. The room-temperature Seebeck coefficient reduces from 576.5 μV K^−1^ for pristine BiCuSeO to 134.3 μV K^−1^ for solely Pb-doped Bi_0.94_Pb_0.06_CuSeO, and finally to 117.7 μV K^−1^ in codoped Bi_0.90_Pb_0.06_Cu_0.96_SeO, on account of the increase in hole concentration (Supplementary Table S1). The temperature-dependent power factors for all Bi_1-x-y_Pb_y_Cu_1-x_SeO compounds are plotted in Fig. 1d. Pristine BiCuSeO displays the lowest power factor among all samples mainly stemming from its extremely low electrical conductivity. For solely dual-vacancy-doped samples, the power factor is slightly improved relative to pristine BiCuSeO. Compared with solely Pb-doped samples, the power factors of dual-vacancy- and Pb- codoped samples are improved in the medium to high temperature range, originating from the increased electrical conductivity together with the considerable Seebeck coefficients. Details of the cause of this phenomenon will be discussed below.

**Figure 2.**
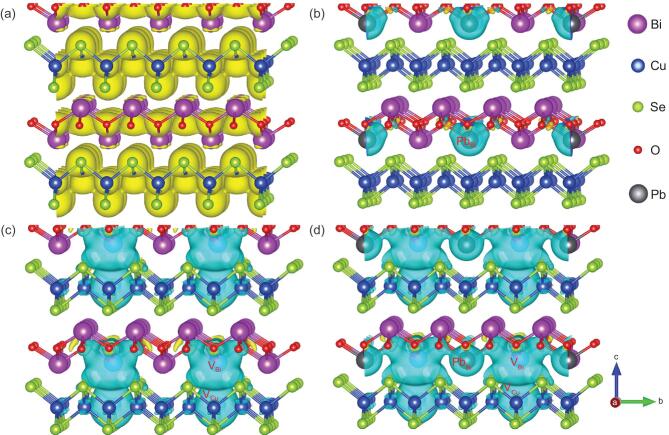
(a) Three-dimensional charge density distribution of pristine BiCuSeO. (b–d) The three-dimensional charge density difference of solely Pb-doped BiCuSeO, solely dual-vacancy-doped BiCuSeO, as well as dual-vacancy- and Pb-codoped BiCuSeO, respectively. The light blue region represents hole accumulation.

To clarify the underlying reasons of the improvement in carrier concentration and electrical conductivity, we calculated the three-dimensional charge density distribution (Fig. 2a) and charge density difference (Fig. 2b–d) for BiCuSeO compounds, respectively. Figure 2a shows the electron charge density distribution of pristine BiCuSeO, where the charges feature a typical localized behavior along the in-plane direction. The three-dimensional charge density differences for the solely Pb-doped BiCuSeO, solely dual-vacancy-doped BiCuSeO, as well as dual-vacancy- and Pb- codoped BiCuSeO are shown in Fig. 2b–d, respectively. After doping Pb at the Bi site, it is clear that the charges are still confined within [Bi_2_O_2_]^2+^ sublayers for Bi_1-y_Pb_y_CuSeO without Bi/Cu dual vacancies (Fig. 2b). Interestingly, for solely Bi/Cu dual-vacancy-doped compound (Fig. 2c), there is a distinct accumulation of holes between the adjacent Bi vacancy and Cu vacancy, signifying noteworthy charge delocalization behavior from [Bi_2_O_2_]^2+^ sublayers to [Cu_2_Se_2_]^2−^ sublayers. The same interlayer delocalization feature of charges is also observed in dual-vacancy- and Pb- codoped material (Fig. 2d), which means that Pb-doping does not destroy the above delocalization behavior. From the above results, it can be concluded that the presence of Bi/Cu dual vacancies is essential for charge delocalization character along the out-of-plane direction. The interlayer charge delocalization character arising from Bi/Cu dual vacancies indicates the existence of interlayer charge-transport channels, which would motivate interlayer charge release. Once there is a charge concentration gradient between the two sublayers, it could be expected that charges trapped in [Bi_2_O_2_]^2+^ sublayers would diffuse into [Cu_2_Se_2_]^2−^ sublayers along the interlayer charge-transport channels bridged by Bi/Cu dual vacancies.

**Figure 3.**
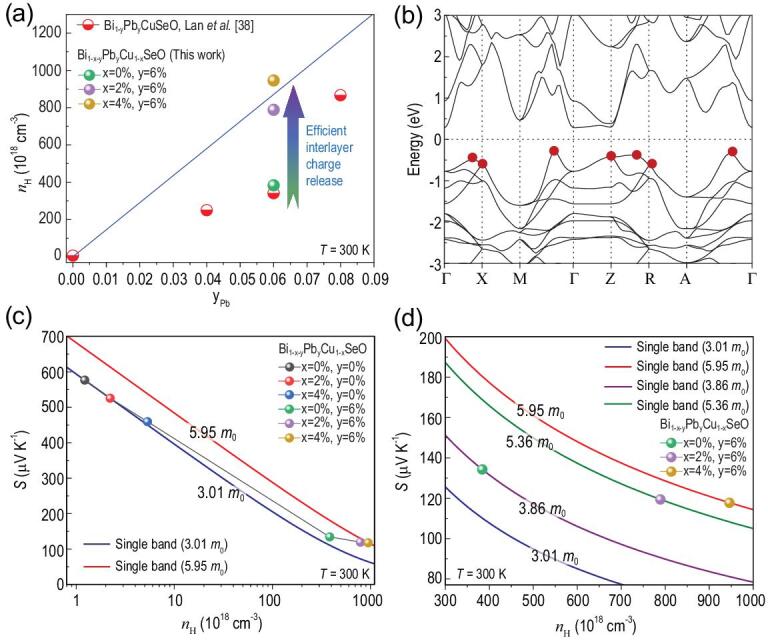
(a) Carrier concentration as a function of Pb content at 300 K with a comparison to literature results [38]. The solid blue line represents the theoretical carrier concentration versus Pb content assuming each Pb atom contributes one hole to the effective hole concentration. (b) Band structure of BiCuSeO. (c) Graphical representation of the Seebeck coefficient as a function of hole concentration (Pisarenko plot) at room temperature. (d) Enlarged Pisarenko plot ranging from 300 × 10^18^ to 1000 × 10^18^ cm^−3^. The solid curves shown in Fig. 3c and d are the calculated Pisarenko curves based on the SPB models with different effective masses of 3.01 *m*_0_ (royal blue line), 3.86 *m*_0_ (purple line), 5.36 *m*_0_ (olive line), and 5.95 *m*_0_ (red line), respectively. With increasing hole concentration, it is clearly seen that the experimental data points deviate significantly from the theoretical Pisarenko curve with higher effective mass value.

In fact, dual vacancies and Pb play different but complementary roles in tailoring the electrical transport performance of BiCuSeO. Specifically, Bi/Cu dual vacancies give rise to delocalized distribution of charges between [Bi_2_O_2_]^2+^ sublayers and [Cu_2_Se_2_]^2−^ sublayers, which offers channels for interlayer charge release. However, although the interlayer delocalization of charges is favorable for the interlayer charge transfer, Bi_1-x_Cu_1-x_SeO material without external dopant lacks sufficient charges for diffusion. Therefore, the increase in the observed carrier concentration is not significant in the solely dual-vacancy-doped samples compared with the pristine BiCuSeO (Supplementary Table S1). On the other hand, upon solely doping external dopant (such as Pb, Ba, Sr and Ca) [32–34,38] at the Bi site, the external dopant can indeed introduce plenty of charges into the charge-reservoir [Bi_2_O_2_]^2+^ sublayers of BiCuSeO material. However, because of the absence of interlayer transport channels, these extrinsic charges are still partially confined within the insulating [Bi_2_O_2_]^2+^ sublayers. The weak interlayer bonding blocks trapped charges from completely diffusing into the conductive [Cu_2_Se_2_]^2−^ sublayers, thereby preventing the trapped charges from becoming conduction carriers. The theoretical hole concentration as a function of Pb content is plotted in Fig. 3a, assuming that each Pb atom (that is acceptor atom) contributes one hole to the effective hole concentration. The experimental carrier concentrations for solely Pb-doped BiCuSeO [38] are significantly lower than the theoretical value, indicating that there is still plenty of room for improvement. Fortunately, dual-vacancy- and Pb- codoped BiCuSeO combines the merits of solely dual vacancy doping and solely Pb doping. Benefiting from the combination of dual-vacancy-induced charge delocalization and extrinsic charges arising from Pb, charge transfer channel and charge concentration gradient exist simultaneously between [Bi_2_O_2_]^2+^ sublayers and [Cu_2_Se_2_]^2−^ sublayers. For dual-vacancy- and Pb- codoped BiCuSeO, the charge concentration gradient triggers release of charges initially trapped in [Bi_2_O_2_]^2+^ sublayers, enabling these charges to diffuse into [Cu_2_Se_2_]^2−^ sublayers along transport channels and thus become conduction carriers. As a result, the carrier concentration is remarkably increased in vacancies/Pb codoped BiCuSeO. As shown in Fig. 3a, the experimental carrier concentrations of vacancies/Pb codoped BiCuSeO deviate strongly from that of the solely Pb-doped BiCuSeO and a theoretical limiting value of ∼8.69 × 10^20^ cm^−3^ is reached, suggesting efficient interlayer charge release in codoped BiCuSeO.

**Figure 4.**
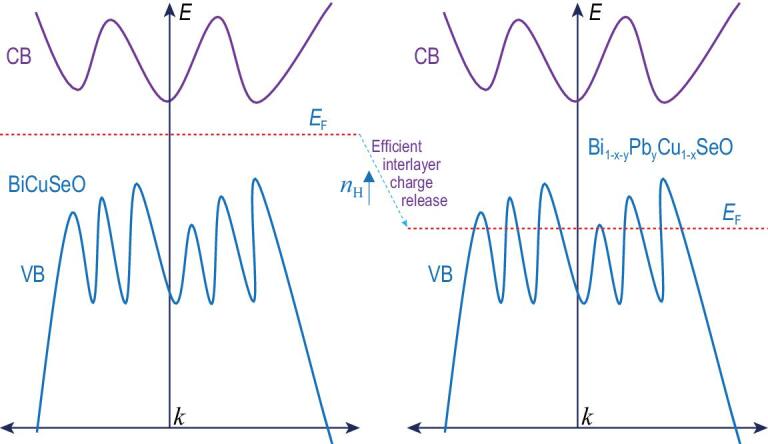
Schematic of Fermi-level movement for Bi_1-x-y_Pb_y_Cu_1-x_SeO versus carrier concentration, indicating multiple converged valence bands involved in the electrical transport process.

To understand the influence of efficient interlayer charge release on the Seebeck coefficient and power factor for Bi_1-x-y_Pb_y_Cu_1-x_SeO, it is necessary to examine the relationship between the Seebeck coefficient and carrier concentration (the so-called Pisarenko plot) at room temperature. Using the single parabolic band (SPB) model [39,40], we calculated Pisarenko curves for different effective masses (Fig. 3c and d; details in the Supplementary data). For pristine BiCuSeO and solely dual-vacancy-doped BiCuSeO (carrier concentration ranges from 1.22 × 10^18^ to 5.32 × 10^18^ cm^−3^), the experimentally observed Seebeck coefficients are mainly located on the solid royal blue line, which indicates an effective mass of 3.01 *m*_0_ (Fig. 3c). For solely Pb-doped sample (Bi_0.94_Pb_0.06_CuSeO, carrier concentration ∼384 × 10^18^ cm^−3^), the measured Seebeck coefficient falls on the theoretical Pisarenko curve with effective mass of 3.86 *m*_0_. For dual-vacancy- and Pb- codoped samples (carrier concentration ranging from 789 × 10^18^ to 946 × 10^18^ cm^−3^), it is remarkable that the experimental Seebeck coefficients gradually deviate to the Pisarenko curve with higher effective mass of 5.95 *m*_0_ (Fig. 3c). Specifically, as can be seen from Fig. 3d, the experimental points of Bi_0.92_Pb_0.06_Cu_0.98_SeO and Bi_0.90_Pb_0.06_Cu_0.96_SeO fall on the Pisarenko plot with different effective masses of 5.36 *m*_0_ (olive line) and 5.95 *m*_0_ (red line), respectively. It can be seen that the effective masses of dual-vacancy- and Pb- codoped BiCuSeO compounds are significantly larger than those of pristine, solely dual-vacancy-doped or solely Pb-doped samples. The increase in effective mass of holes is closely related to the multiple valence bands of BiCuSeO [15,41,42]. As shown in Fig. 3b, the first-principles simulations for the electronic band structure of BiCuSeO indicate complex multiband valence states that lie near each other in energy. The efficient interlayer charge release from [Bi_2_O_2_]^2+^ sublayers into [Cu_2_Se_2_]^2−^ sublayers in real space endows dual-vacancy- and Pb- codoped BiCuSeO with drastically increased carrier concentration. Correspondingly, with increasing carrier concentration, in reciprocal space [7] the Fermi level is pushed into the valence band and more hole pockets are populated with hole carriers [3] for the p-type dual-vacancy- and Pb- codoped BiCuSeO (see Fig. 4). The activated multiple converged valence bands account for the increase in effective mass, which is thought to be responsible for the increase in the Seebeck coefficient and the associated power factor at the similar carrier concentration.

**Figure 5.**
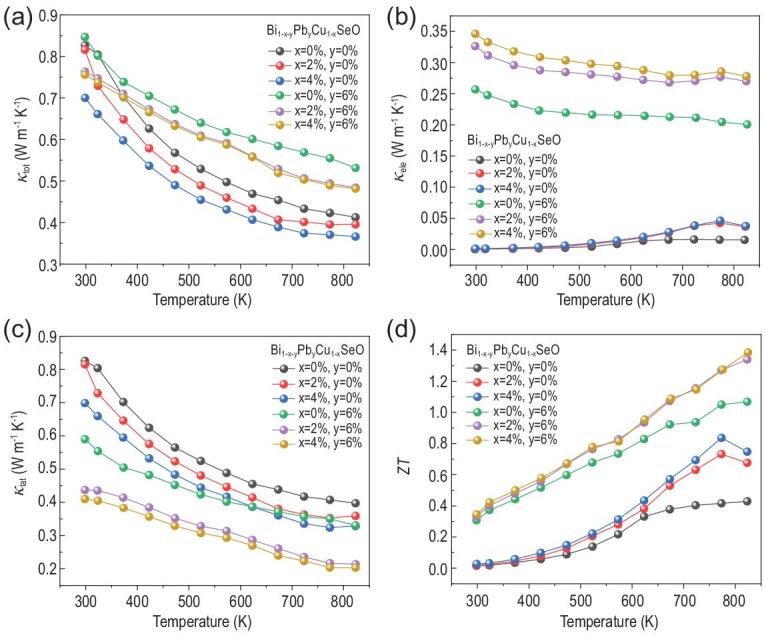
Total thermal conductivity (a), electronic thermal conductivity (b), lattice thermal conductivity (c), and dimensionless figure of merit *ZT* (d) as a function of temperature for Bi_1-x-y_Pb_y_Cu_1-x_SeO.

The total thermal conductivities *κ*_tot_, the electronic thermal conductivities *κ*_ele_ and the lattice thermal conductivities *κ*_lat_ as a function of temperature for Bi_1-x-y_Pb_y_Cu_1-x_SeO samples are plotted in Fig. 5a–c, respectively. The electronic thermal conductivity *κ*_ele_ is calculated by the Wiedemann-Franz law *κ*_ele_*= LσT*, where *L* is the Lorenz number, *σ* is the electrical conductivity, and *T* is the absolute temperature [43]. Herein, the *L* value was estimated from the SPB model with acoustic phonon scattering [44–47] (details in the Supplementary data). The lattice part of thermal conductivity *κ*_lat_ was obtained by subtracting the electronic part from the total thermal conductivity. For samples solely doped with Bi/Cu dual vacancies, the electronic thermal conductivity accounts for a very low percentage of the total thermal conductivity because of the low electrical conductivity (Fig. 5b). Meanwhile, both the total thermal conductivity and the lattice thermal conductivity decrease with increasing concentration of Bi/Cu dual vacancies. This is because vacancies have the capacity to enhance phonon scattering, which reduces the mean free path of low-frequency heat-carrying phonons [47]. Upon doping with Pb for the samples containing Bi/Cu dual vacancies, as a result of the much increased carrier concentration and enhanced electrical conductivity, the electronic thermal conductivity of dual-vacancy- and Pb- codoped BiCuSeO samples is obviously increased in comparison with that of solely dual-vacancy-doped or Pb-doped samples (Fig. 5b). Where the content of point defects in Bi_1-x-y_Pb_y_Cu_1-x_SeO is gradually increased after doping dual vacancies and Pb, the lattice thermal conductivity for codoped BiCuSeO is significantly reduced arising from the enhancement in phonon scattering. The minimum lattice thermal conductivity of 0.2 W m^−1^ K^−1^ is obtained for the Bi_0.90_Pb_0.06_Cu_0.96_SeO compound at 823 K (Fig. 5c), which compensates for the increase in electronic thermal conductivity, and ensures a very low total thermal conductivity in the codoped BiCuSeO.

Combining the electrical and thermal transport properties, the figure of merit *ZT* as a function of temperature for Bi_1-x-y_Pb_y_Cu_1-x_SeO samples are plotted in Fig. 5d. The dual-vacancy- and Pb- codoped BiCuSeO combines the merits of solely dual-vacancy-doping and solely Pb-doping, that is the figure of merit *ZT* values are enhanced over the entire test temperature range. Because of the high power factor of ∼8.1 μW cm^−1^ K^−2^ coupling with the low total thermal conductivity of ∼0.5 W m^−1^ K^−1^, a maximum *ZT* value of ∼1.4 is obtained at 823 K in the codoped Bi_0.90_Pb_0.06_Cu_0.96_SeO, which is about 250% higher than that of the pristine BiCuSeO (*ZT* ∼ 0.4), 100% higher than that of the solely dual-vacancy-doped Bi_0.96_Cu_0.96_SeO (*ZT* ∼ 0.7), and 40% higher than that of the solely Pb-doped Bi_0.94_Pb_0.06_CuSeO (*ZT* ∼ 1.0). Combined with the aforementioned discussions, the notable enhancement in the thermoelectric figure of merit is unambiguously correlated to the efficient interlayer charge release effect caused by Bi/Cu dual vacancies and Pb codoping.

## CONCLUSION

In conclusion, we present a promising strategy for activating multiple Fermi pockets and optimizing the thermoelectric properties in the BiCuSeO system by means of efficient interlayer charge release. This type of efficient charge release is realized by constructing channels for interlayer charge-transport process and providing plenty of extrinsic charges to diffuse along these channels. The efficient interlayer charge release produces substantial enhancement of carrier concentration while maintaining a considerable Seebeck coefficient as the released carriers activate multiple converged valence bands. Benefiting from the combination of improved power factor and low thermal conductivity, a significant enhanced *ZT* value of ∼1.4 is achieved in Bi_0.90_Pb_0.06_Cu_0.96_SeO at 823 K. The present strategy could be applied to other materials with layered structures and could inject fresh energy into the field of thermoelectric studies.

## METHODS

The experimental details are given in the Supplementary data.

## Supplementary Material

nwaa085_Supplement_File
