# Supplementary material for: Efficient interlayer charge release for high-performance layered thermoelectrics
Source: Natl Sci Rev. 2020 Apr 28;8(2):nwaa085. doi: 10.1093/nsr/nwaa085 (PMC8288408; doi:10.1093/nsr/nwaa085)
Supplement: nwaa085_Supplement_File [file nwaa085_supplement_file.doc]

Supplementary Information

Efficient Interlayer Charge Release for High-Performance Layered Thermoelectrics

Hao Zhu^1^, Zhou Li^1^, Chenxi Zhao^1^, Xingxing Li^3^, Jinlong Yang^3^, Chong Xiao^1,2*^ and Yi Xie^1,2*^

1 Hefei National Laboratory for Physical Sciences at the Microscale, University of Science and Technology of China, Hefei, Anhui 230026, P. R. China

2 Institute of Energy, Hefei Comprehensive National Science Center. Hefei, Anhui 230031, P. R. China

3 Department of Chemical Physics, Hefei National Laboratory for Physical Sciences at the Microscale and Synergetic Innovation Center of Quantum Information & Quantum Physics, University of Science and Technology of China, Hefei, Anhui 230026, P. R. China

Corresponding Author

* *cxiao@ustc.edu.cn; yxie@ustc.edu.cn*

Experimental Section

*Synthesis and Densification*: Polycrystalline samples with the nominal compositions of Bi_1-x-y_Pb_y_Cu_1-x_SeO (x=0, 0.02, 0.04; y=0, 0.06) were synthesized by a solid-state reaction route combined with the hot-pressing process as described below. High-purity Bi (4N), Bi_2_O_3_ (4N), Cu (4N), Se (4N), and Pb (5N) powders were thoroughly mixed and ground using an agate mortar and pestle in an argon-filled glove box, and subsequently sealed in evacuated quartz tubes. The loaded tubes were slowly heated to 573 K, soaked at this temperature for 12 h, then to 973 K, soaked there for 10 h, and subsequently cooled slowly to room temperature. The resultant ingots were hand crushed into fine powders in an argon atmosphere, which were densified using an induction-heating hot press system under an axial compressive stress of 80 MPa at 923 K for 30 min in vacuum to obtain highly dense disk-shaped pellets.

*X-ray Diffraction and Electron Microscopy*: The phase composition of the obtained powder samples was characterized by using the powder X-ray diffraction analysis (PXRD; TTR-III, Rigaku, Japan) with Cu K*α* (*λ* = 1.5418 Å) radiation. The high-angle annular dark-field scanning transmission electron microscopy (HAADF-STEM) images and STEM EDS element mapping were characterized by using a spherical aberration-corrected field-emission transmission electron microscope (JEOL ARM-200F, Japan) equipped with an energy-dispersive spectrometer.

*Electrical Properties*: The obtained hot-pressed pellets were cut and polished into bars with dimensions 2 × 3 × 10 mm^3^ for electrical measurement. The electrical conductivity and Seebeck coefficient were measured simultaneously under a low-pressure helium atmosphere from room temperature to 823 K using a CTA instrument (Cryoall, China).

*Thermal Conductivity*: The obtained hot-pressed pellets were polished into disks with a diameter of ~12.7 mm and a thickness of ~2.1 mm for thermal diffusivity measurements in the range from room temperature to 823 K using a laser flash technique (LFA457, Netzsch, Germany). The total thermal conductivity was calculated from *κ*_tot_ = *DdC*_p_, where *D*, *d*, and *C*_p_ represent the thermal diffusivity coefficient, sample density, and specific heat capacity, respectively. The specific heat capacity was obtained by the differential scanning calorimeter method in the range 300-823 K (DSC Q2000, Netzsch, Germany), and the density was determined using the dimensions and mass of the sample.

*Hall Measurements*: The carrier concentrations were measured by the Van der Pauw method using the AC field Hall effect measurement option installed on the standard Model 8404 (Lake Shore 8400 series, USA).

*Density functional theory (DFT) calculations*: First principles calculations are performed with the vdW density functional SCAN+rVV10,^[1]^ which combines the strongly constrained and appropriately normed (SCAN)^[2]^ meta-GGA and the revised Vydrov-van Voorhis nonlocal correction functional (rVV10),^[3]^ as implemented in Vienna ab initio simulation package (VASP).^[4]^ The projector augmented wave (PAW) potential^[5]^ and the plane-wave cut-off energy of 520 eV are adopted. Both the lattice constants and positions of all atoms are relaxed until the force is less than 0.01 eV/Å. The criterion for the total energy is set as 1×10^-6^ eV.

**Calculation of Lorenz number and effective mass**

For typical thermoelectric materials, most of which are degenerate semiconductors.^[6]^ According to the single band assumption, the Lorenz number *L* and effective mass *m** can be expressed as follow: ^[7-9]^

$S=\pm\frac{k_{B}}{e}\left(\frac{\left(r+5/2\right)F_{r+3/2}(\eta)}{(r+3/2)F_{r+1/2}(\eta)}-\eta\right)$, (1)

, (2)

$F_{i}(\xi)=\int_{0}^{\infty} \frac{x^{i}dx}{1+e^{x-\xi}}$, (3)

$L=\left(\frac{k_{B}}{e}\right)^{2}\left(\frac{(r+7/2)F_{r+5/2}(\eta)}{(r+3/2)F_{r+1/2}(\eta)}-\left[\frac{(r+5/2)F_{r+3/2}(\eta)}{(r+3/2)F_{r+1/2}(\eta)}\right]^{2}\right)$, (4)

In the above equations, *S* is the measured Seebeck coefficients, *k_B_* is the Boltzmann constant, *e* is the electron charge, *η* = *E_F_ / k_B_T* is the reduce Fermi energy where *E_F_* is the electron Fermi level measured upward from the band edge, *n* is the carrier concentration, *ħ* is the reduced Planck constant, *F*_i_ (*ζ*) is the Fermi integrals, *r* is the scattering parameter. Specifically, when acoustic phonon scattering is regarded as the primary carrier scattering mechanism, the value corresponding to *r* is −1/2. The effective mass and Lorenz number can be calculated from **Equation** (1) ~ (4).

**Table S1.** Electrical conductivity *σ*, Seebeck coefficient *S*, carrier concentration *n*_H_, reduced Fermi levels *ξ*_F_, and Lorenz constants *L* at room temperature for Bi_1-x-y_Pb_y_Cu_1-x_SeO.

| Samples | *σ* (S cm^-1^) | *S* (μV/K) | *n*_H (_10^18^ cm^-3^) | *ξ*_F_ | *L* (10^-8^ W Ω K^-1^) |
| --- | --- | --- | --- | --- | --- |
| x=0%, y=0% | 1.27 | 576.51 | 1.22 | -4.67 | 1.491 |
| x=2%, y=0% | 1.84 | 525.48 | 2.22 | -4.08 | 1.492 |
| x=4%, y=0% | 3.05 | 459.49 | 5.32 | -3.31 | 1.496 |
| x=0%, y=6% | 483.75 | 134.28 | 384 | 1.39 | 1.779 |
| x=2%, y=6% | 596.61 | 119.37 | 789 | 1.79 | 1.833 |
| x=4%, y=6% | 629.89 | 117.73 | 946 | 1.84 | 1.840 |

**Table S2**. Room-temperature densities of samples included in the study.

| Samples  Bi_1-x-y_Pb_y_Cu_1-x_SeO | Measured density  (g cm^-3^) | Theoretical density  (g cm^-3^) | Relative density  (%) |
| --- | --- | --- | --- |
| x=0%, y=0% | 8.41 | 8.90 | 94.47 |
| x=2%, y=0% | 8.38 | 8.90 | 94.10 |
| x=4%, y=0% | 8.35 | 8.90 | 93.85 |
| x=0%, y=6% | 8.48 | 8.90 | 95.27 |
| x=2%, y=6% | 8.43 | 8.90 | 94.74 |
| x=4%, y=6% | 8.34 | 8.90 | 93.71 |


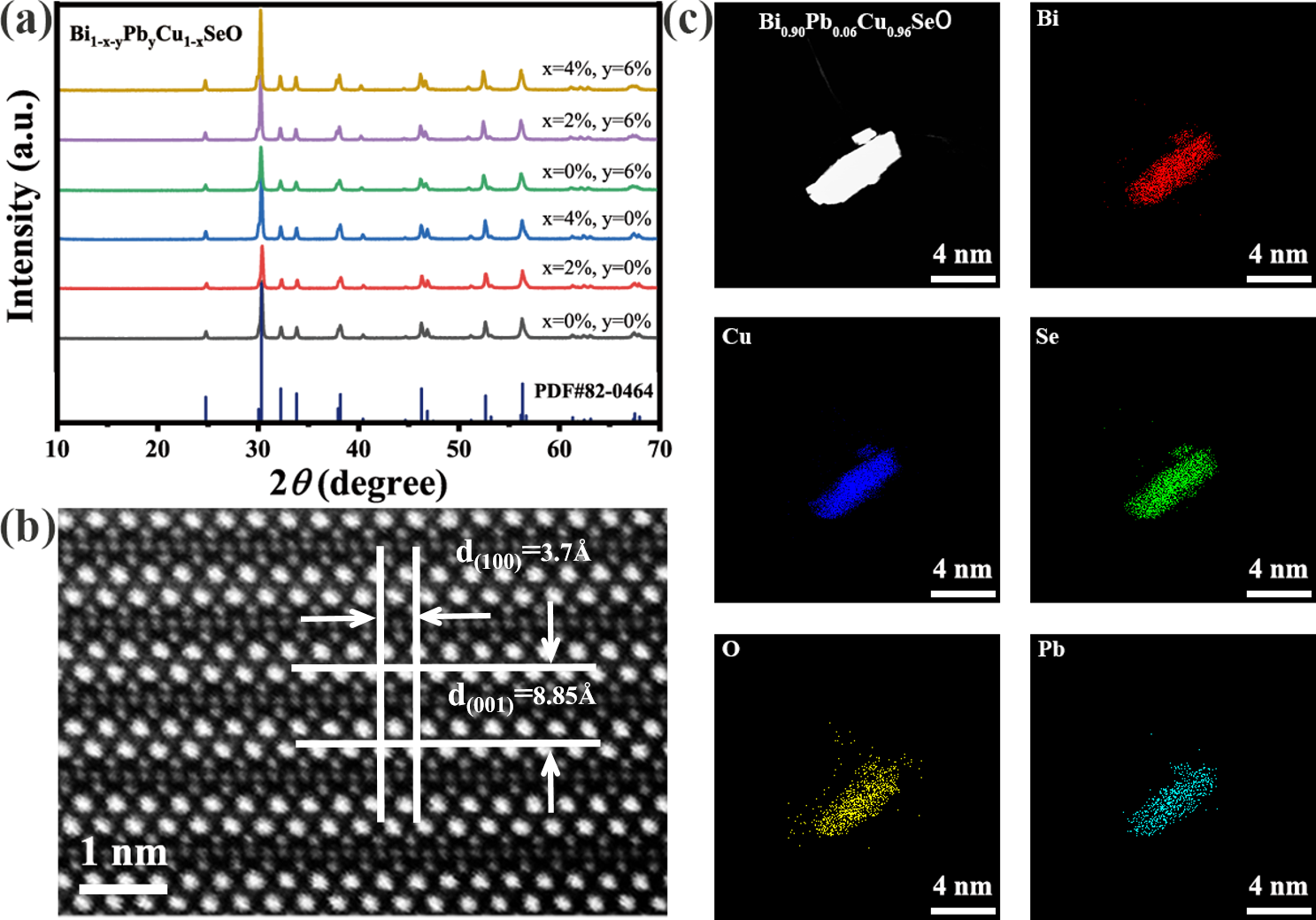


**Figure S1.** (a) Powder XRD patterns for Bi_1-x-y_Pb_y_Cu_1-x_SeO (x=0, 0.02, 0.04, y=0, 0.06) samples, (b) HAADF-STEM image of Bi_0.90_Pb_0.06_Cu_0.96_SeO along the [100] direction, and (c) STEM-EDS elemental mapping images of Bi_0.90_Pb_0.06_Cu_0.96_SeO.


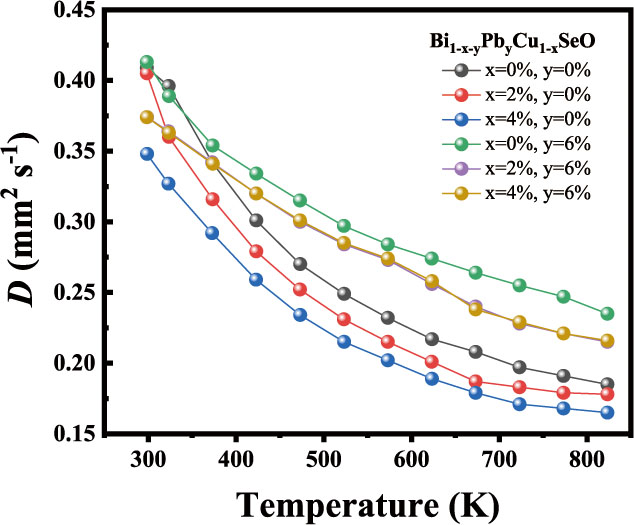


**Figure S2.** Thermal diffusivity coefficient as a function of temperature for Bi_1-x-y_Pb_y_Cu_1-x_SeO.

**
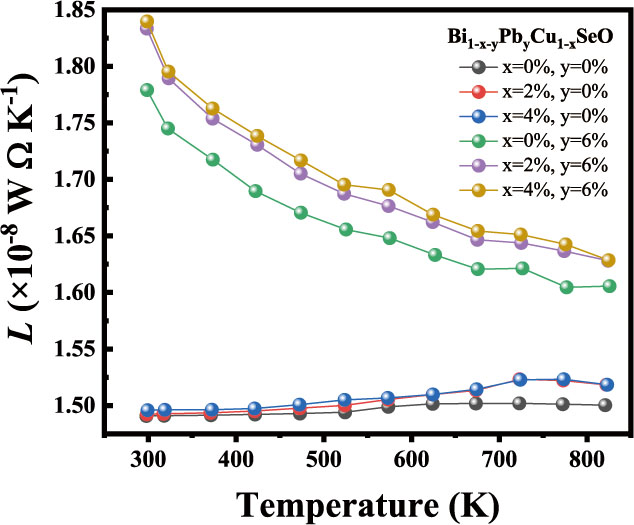
**

**Figure S3.** Temperature-dependent Lorenz number for Bi_1-x-y_Pb_y_Cu_1-x_SeO.

**REFERENCES**

1. Peng H, Yang ZH and Perdew JP *et al*. Versatile van der Waals density functional based on a meta-generalized gradient approximation. *Phys Rev X* 2016; **6**: 041005.
2. Sun J, Ruzsinszky A and Perdew JP. Strongly constrained and appropriately normed semilocal density functional. *Phys Rev Lett* 2015; **115**: 036402.
3. Vydrov OA and Van Voorhis T. Nonlocal van der Waals density functional: The simpler the better. *J Chem Phys* 2010; **133**: 244103.
4. Kresse G and Furthmüller J. Efficient iterative schemes for ab initio total-energy calculations using a plane-wave basis set. J. *Phys Rev B* 1996; **54**: 11169-86.
5. Blöchl PE. Projector augmented-wave method. *Phys Rev B* 1994; **50**: 17953-79.
6. Snyder GJ and Toberer ES. Complex thermoelectric materials. *Nat Mater* 2008; **7**: 105-14.
7. Lan JL, Liu YC and Zhan B *et al*. Enhanced thermoelectric properties of Pb-doped BiCuSeO ceramics. *Adv Mater* 2013; **25**: 5086-90.
8. Tan G, Zhao LD and Shi F *et al*. High thermoelectric performance of p-type SnTe via a synergistic band engineering and nanostructuring approach. *J Am Chem Soc* 2014; **136**: 7006-17.
9. Zhao LD, Lo SH and He J *et al*. High performance thermoelectrics from earth-abundant materials: enhanced figure of merit in PbS by second phase nanostructures. *J Am Chem Soc* 2011; **133**: 20476-87.
